# Utilization of the neighborhood design to evaluate suitable cover crops and their density for *Echinochloa colona* management

**DOI:** 10.1371/journal.pone.0254584

**Published:** 2021-07-12

**Authors:** Amar Matloob, Bhagirath Singh Chauhan

**Affiliations:** 1 Department of Agronomy, MNS University of Agriculture, Multan, Pakistan; 2 Queensland Alliance for Agriculture and Food Innovation (QAAFI) and School of Agriculture and Food Sciences (SAFS), The University of Queensland, Gatton, Queensland, Australia; 3 Chaudhary Charan Singh Haryana Agricultural University (CCSHAU), Hisar, Haryana, India; University of Minnesota, UNITED STATES

## Abstract

Summer weed species, including *Echinochloa colona*, are becoming problematic in the eastern grain region of Australia, but cover crops can be useful to suppress weeds during the summer fallow period. The present study evaluated the growth and seed production of *E*. *colona* grown alone or with four and eight cover crop plants per pot (i.e., 80 and 160 plants m^-2^). Four legume (cowpea, lablab, pigeonpea, and soybean) and two grass (forage sorghum and Japanese millet) cover crops were used. Interference by cover crops reduced the height, the number of leaves and tillers, inflorescence number, seed production, and biomass of this weed than when it was grown alone. Cover crops differed in their ability to suppress the growth and seed production of *E*. *colona*. The effect of cover crop density on the studied attributes was non-significant in most cases. Pigeonpea as a cover crop was the least effective in suppressing the growth and seed production of *E*. *colona*. In general, leguminous cover crops exhibited less suppression of *E*. *colona* than grasses. Forage sorghum was most efficient in reducing the growth of this weed. Forage sorghum and Japanese millet reduced *E*. *colona* leaf and tiller numbers per plant by 90 and 87%, respectively. These cover crops reduced *E*. *colona* leaf number to only 17 per plant as against 160 per plant recorded without cover crops. Inflorescence number per *E*. *colona* plant growing alone was as high as 48. However, it was reduced by 20–92% when this weed was grown with cover crop plants. *E*. *colona*’s seed production was significantly suppressed by all the cover crops, except pigeonpea. Biomass of *E*. *colona* was suppressed largely by forage sorghum and Japanese millet compared to other cover crops. Among the cover crops, pigeonpea produced the lowest biomass of 11 g pot^-1^, and the highest biomass (114 g pot^-1^) was produced by forage sorghum. The study demonstrated the usefulness of cover crops, especially forage sorghum and Japanese millet, to suppress the growth and seed output of *E*. *colona*.

## Introduction

*Echinochloa colona* (L.) Link is a problematic annual C_4_ grass weed of 35 cropping systems in more than 60 countries globally, especially in tropical and subtropical regions of Asia, Africa, and Australia [1]. In northern cropping systems of Australia, *E*. *colona* has been recognized as an important summer annual weed [2, 3]. High competitive ability, relative growth rate, dry matter accumulation, and seed output, profuse tillering, early flower bud initiation, and allelopathic inhibitory activity make this weed troublesome and noxious [1, 4–6]. Overreliance on glyphosate as a sole means to control this weed in summer fallows has led to the evolution of herbicide-resistant biotypes of *E*. *colona* in the USA, Argentina, and Australia [7]. The largest area under glyphosate-resistant *E*. *colona* occurs in three Australian states, i.e., New South Wales, Queensland, and Western Australia. In Australia, the first case of glyphosate-resistant *E*. *colona* was reported from New South Wales in 2007. Subsequently, resistant biotypes were reported from Queensland and Western Australia during 2009 and 2010, respectively [7]. Estimates indicate that infestation of crop fields with glyphosate-resistant grasses, including *E*. *colona*, will increase the cost incurred in controlling weeds by AU$ 40–90 ha^-1^ per year [8]. Economic and ecological costs associated with herbicides have compelled alternative weed management options in agro-ecosystems that are cost-effective and environmentally benign.

Integrated weed management aims is to maintain the weed population at a manageable level while preventing weed flora shifts and resistance evolution in weeds. The impact of weed competition can be reduced to a significant extent by manipulation of crop row orientation, row spacing, planting time and density, and weed suppressive cover crops [9, 10] due to early season canopy closure [11, 12]. The weed suppression potential of a cover crop is reflected as a reduction in endemic weed cover [13] and dry biomass [14].

The use of cover crops has been suggested as an effective approach to overcome herbicide-related ill effects in cropping systems [14–16]. Cover crops offer alternative soil and weed management options to tillage and chemical weed control [17, 18]. Additional benefits of cover crops are reductions in soil erosion and improvement of soil health, nutrient and water relations, provision of nectar for pollinators, and habitat for beneficial insects. Weed suppression is one of the key services rendered by cover crops in cropping systems. The magnitude of weed suppression is governed by cover crop species, planting density, biomass of living cover crop stand or decomposing tissues, concentration of allelochemicals released by cover crops, weed species, and other management interventions [14, 16, 19]. Cover crops suppress weeds by posing physical (smothering, acquisition of water and nutrients otherwise destined for weed plants, reduced light transmission, modification of soil temperatures, and mulch) and chemical (release of allelochemicals by living and decomposing plant tissues) effects and attenuating environmental cues [14, 19].

To achieve optimal weed control, plant species chosen as a cover crop need careful consideration [19, 20]. Currently, there is limited information on the contribution of contrasting cover crops to weed suppression and crop productivity [16]. Compared to Europe and the USA, there is limited information available on the effect of cover crops on weed suppression in Australia. For effective utilization, cover crop species and their densities need to be optimized. Hence, there is a need to document the response of different cover crops and their varying densities on *E*. *colona* growth and reproductive attributes.

The target-neighbor design can be helpful to evaluate suitable cover crops and their densities since it allows to specifically appraise the response of fixed density of a focal species to varying densities of associate species [21, 22]. Individual plant-centered experimental designs are useful to ascertain interactions between target plants and their neighboring plants by switching the focus from population to individual responses of target plants [23]. Such methods are relevant especially when interest is to unravel the role of spatial arrangements of the crop-weed community in modifying the competitive outcomes [22, 24]. This would enable to devise of planting patterns that can avert weed growth effectively. The present study was undertaken to evaluate the effect of different cover crops on the growth and seed production of *E*. *colona* following the target-neighborhood design. Another objective was to ascertain the density-specific weed suppression by cover crops. The hypotheses tested in this study were: (i) weed suppressive ability will differ between leguminous and grassy cover crops, and (ii) increasing cover crop density will suppress *E*. *colona* growth and reproduction more efficiently.

## Material and methods

Seeds of *E*. *colona* were collected from the Gatton research fields of the University of Queensland, Queensland, Australia (approximately latitude 27.33° S, longitude 152.16° E and altitude 94 m a.s.l.) in 2018. Plastic pots (25 cm diameter and 30 cm height) were filled with potting mix (pH 5.6 and electrical conductivity 1.6 dS m^-1^; Centenary Landscaping Supplies, Queensland, Australia) and placed in a screenhouse. Three seeds of *E*. *colona* were sown in the center of each pot either alone or with four or eight cover crop plants to quantify their response to interference. One healthy seedling of the weed was maintained per pot after thinning within 10 days after sowing (DAS). Cover crops and their respective cultivars used in the present study were: cowpea [*Vigna unguiculata* (L.) Walp.; cv. Caloona], forage sorghum [*Sorghum bicolor* (L.) Moench; cv. Betta Graze], Japanese millet [*Echinochloa esculenta* (A.Braun) H.Scholz; cv. Shirohie], lablab [*Lablab purpureus* (L.) Sweet; cv. Highworth], pigeonpea [*Cajanus cajan* (L.) Millsp.; an advance breeding line PC-1] and soybean [*Glycine max* (L.) Merr.; cv. Richmond]. The cover crop densities of four and eight plants per pot (corresponding to 80 and 160 plants m^-2^, respectively) were maintained by sowing seeds of the respective cover crop as per treatment equidistant from each other. The cover crop seeds were sown at a depth of 3 cm and a distance of 11 cm from *E*. *colona* seeds. The selected cover crop densities represent different levels of shading caused by cover crops after canopy closure. The weed and cover crop plants emerged within 6–8 DAS. The pots were irrigated daily using an automated irrigation system so that moisture was not limiting. The experimental pots were placed at a distance of 50 cm from each other and moved to a new position weekly to avoid any position effect.

The effect of cover crop interference on *E*. *colona* growth and seed production was quantified by measuring plant height, and the number of leaves, tillers, inflorescence, and seeds at 56 DAS. *E*. *colona* plant height was measured from the base of the plant to the tip of the uppermost leaf. The study was terminated at 56 DAS when the lower leaves of *E*. *colona* started turning yellowish. At harvest, the number of inflorescences and seeds plant^-1^ for *E*. *colona* was counted. The aboveground biomass of *E*. *colona* was measured after drying the harvested plant samples in an oven at 70°C for 48 h. Height and aboveground biomass of cover crops were measured at harvest. No insect attack or disease incidence was observed during the study period, and hence no curative measures were undertaken.

The study was conducted using a completely randomized design with four replications and there were three experimental runs from September 2019 to April 2020. A new experimental run was initiated within a month of termination of the previous run. Before analyses, the homogeneity and normality of data were checked, and analysis of variance (ANOVA) was performed using GenStat (19^th^ edition; VSN International, Hemel Hempstead, UK). Data were pooled across the runs (a total of 12 replications) for further statistical analyses as no significant interaction between treatments and experimental runs were observed. Differences amongst treatment means were evaluated by Fisher’s protected Least Significant Differences (LSD, p≤0.05) test.

## Results

### Morphological attributes

**Fig 1:**
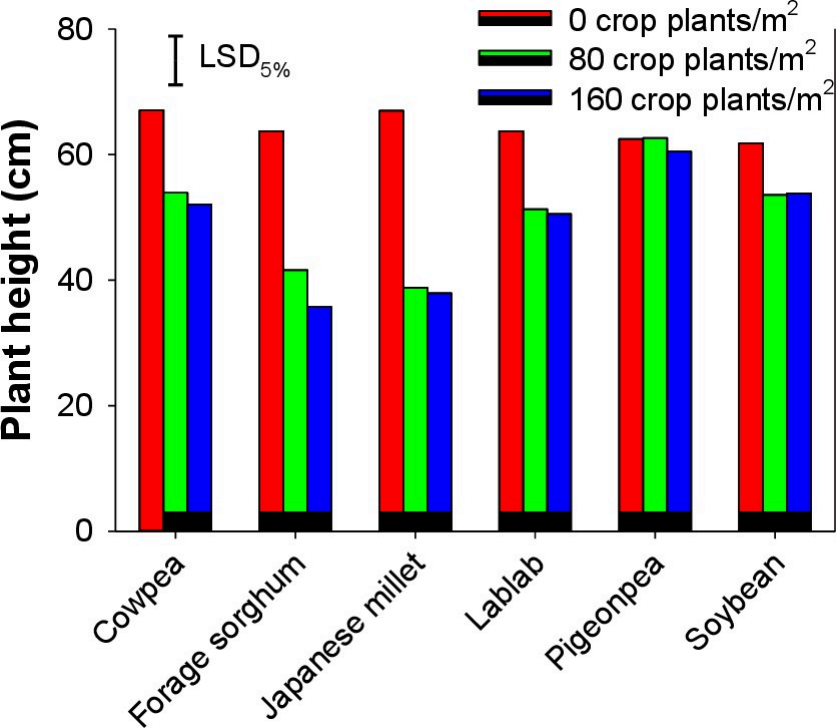
Plant height of *Echinochloa colona* when it was grown alone or in interference with 80 and 160 plants m^-2^ of various cover crops.

*Echinochloa colona* plant height was significantly affected by the type of cover crop; however, it was not affected by cover crop densities (i.e., 80 and 160 plants m^-2^) (Fig 1). The height was taller in pots where *E*. *colona* plants were growing alone. Irrespective of their densities, leguminous cover crops did not reduce the plant height of *E*. *colona* and pigeonpea was least effective in this regard. Forage sorghum and Japanese millet at both densities suppressed the height of *E*. *colona* by 35–44% and 42–43%, respectively, over individually growing *E*. *colona* plants.

**Fig 2:**
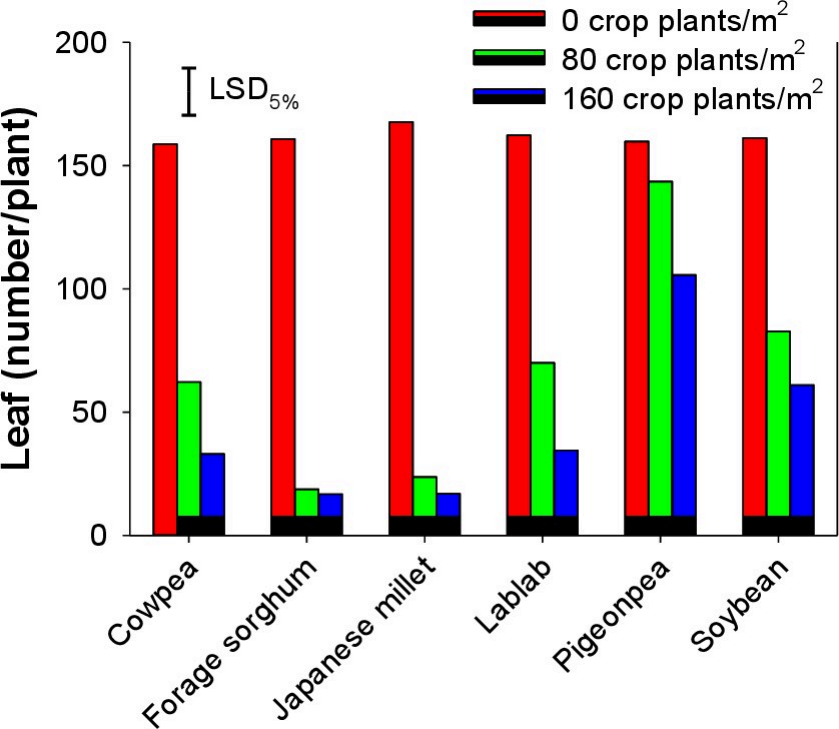
Leaf number of *Echinochloa colona* when it was grown alone or in interference with 80 and 160 plants m^-2^ of various cover crops.

A significant reduction in the leaf number of *E*. *colona* was noticed when this weed was grown in interference with cover crops (Fig 2). Leaves per plant were more in pots where *E*. *colona* was grown alone. Leaf number of *E*. *colona* did not vary when grown in interference with 80 pigeonpea plants m^-2^. However, increasing pigeonpea density from 80 to 160 plants m^-2^ significantly reduced the number of leaves of *E*. *colona*. Without cover crop interference, *E*. *colona* produced as much as 168 leaves plant^-1^. Interestingly, cover crops like forage sorghum and Japanese millet reduced *E*. *colona* leaves plant^-1^ by 90%. The mean value declined to only 17 leaves plant^-1^ when grown with 160 plants m^-2^ (Fig 2).

**Fig 3:**
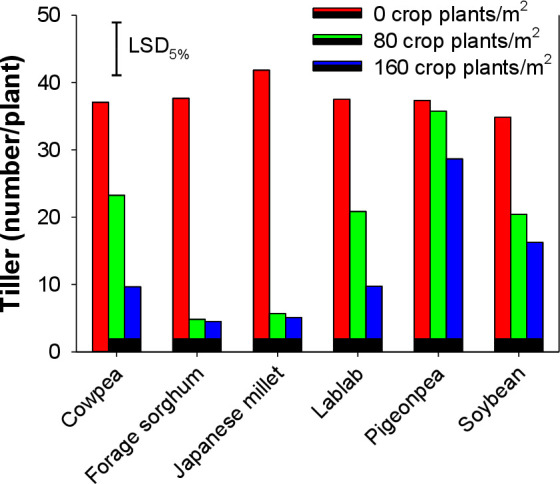
Tiller number of *Echinochloa colona* when it was grown alone or in interference with 80 and 160 plants m^-2^ of various cover crops.

Tillers were fewer when *E*. *colona* plants were grown in interference with cover crops. The presence of cover crop plants in the vicinity suppressed the tillering ability of *E*. *colona* (Fig 3). Increasing the density of cover crops had no significant effect on the number of tillers of *E*. *colona* except for cowpea and lablab. Increasing density from 80 to 160 plants m^-2^ increased the magnitude of the reduction from 37 and 44%, respectively, to 74% in both cases. Forage sorghum and Japanese millet as a cover crop suppressed tillering of *E*. *colona* by 87% compared with tiller numbers of *E*. *colona* plants grown alone.

### Inflorescence number and seed production

**Fig 4:**
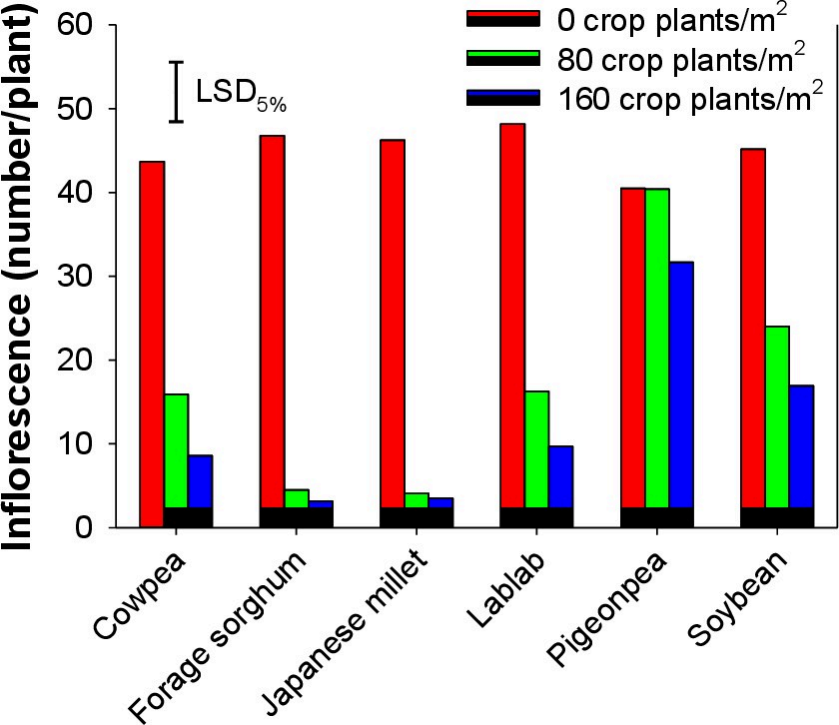
Inflorescence number of *E*. *colona* when it was grown alone or in interference with 80 and 160 plants m^-2^ of various cover crops.

**Fig 5:**
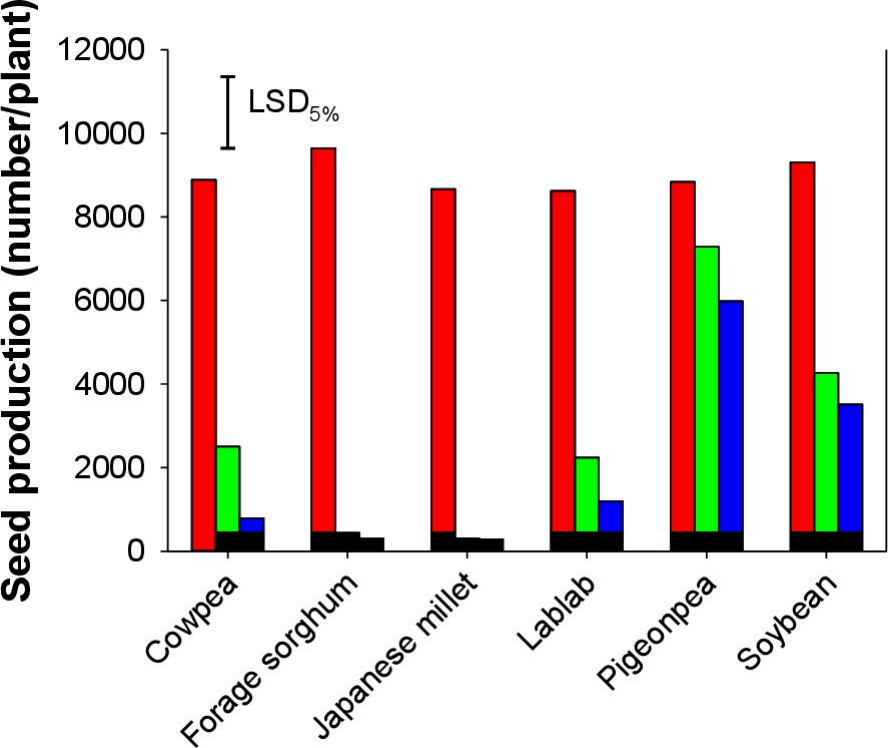
Seed production by *Echinochloa colona* when it was grown alone or in interference with 80 and 160 plants m^-2^ of various cover crops.

Interference by cover crops reduced the number of inflorescences (Fig 4) and seed production (Fig 5) of *E*. *colona*. *E*. *colona* plants growing alone produced 48 inflorescences plant^-1^. However, a 20–92% reduction in inflorescence number was recorded when *E*. *colona* plants were grown with cover crop plants. Increasing the density of cover crops in experimental units had no significant effect on the inflorescence number of *E*. *colona* (Fig 4). Pigeonpea at 80 plants m^-2^ was ineffective in causing any reduction in the inflorescence number. Compared to legume cover crops, grasses such as forage sorghum and Japanese millet were efficient in reducing the inflorescence number of *E*. *colona* (Fig 4).

Plants of *E*. *colona* grown alone produced 8165 to 10310 seeds plant^-1^ (Fig 5). However, seed production significantly declined in the presence of cover crops. Averaged across cover crops, *E*. *colona* produced 2840 and 2010 seeds plant^-1^ in interference with 80 to 160 plants m^-2^ of cover crops against a mean value of 9080 seeds plant^-1^ when grown without cover crop interference (Fig 5). However, the cover crop density-related reduction in seed production of *E*. *colona* was non-significant. Although all the cover crops significantly reduced the reproductive output of *E*. *colona*, yet a greater reduction was found when *E*. *colona* was grown in interference with forage sorghum and Japanese millet. The presence of pigeonpea as a neighboring cover crop had a minimal effect on the seed production of *E*. *colona* (Fig 5).

### Weed biomass

**Fig 6:**
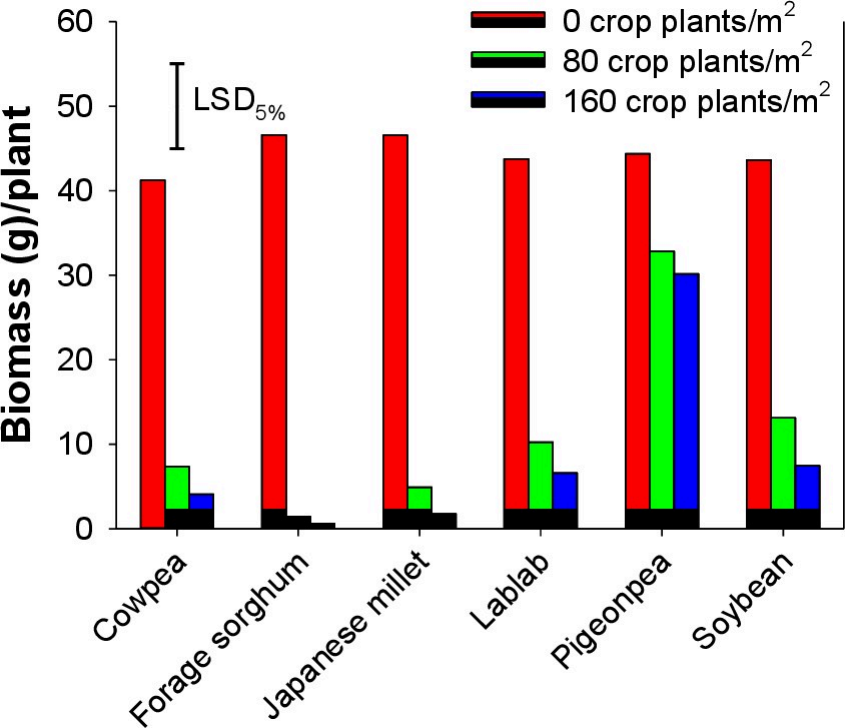
Biomass of *Echinochloa colona* when it was grown alone or in interference with 80 and 160 plants m^-2^ of various cover crops.

Biomass of *E*. *colona* growing alone ranged from 41.2 to 48.6 g plant^-1^ with a mean value of 44.7 g plant^-1^ averaged across all control plants. This value was as low as 8.3 g and 5.1 g plant^-1^ when *E*. *colona* was grown in interference with 80 to 160 plants m^-2^ of cover crops, respectively (Fig 6). Among cover crops, pigeonpea was the least effective in suppressing the biomass of *E*. *colona*. It was followed by soybean, lablab, and cowpea as cover crops resulting in the next higher biomass of *E*. *colona*. Maximum *E*. *colona* biomass suppression was recorded for forage sorghum and Japanese millet. Except for pigeonpea, all cover crops resulted in similar suppression of *E*. *colona* biomass.

### Cover crop growth

**Fig 7:**
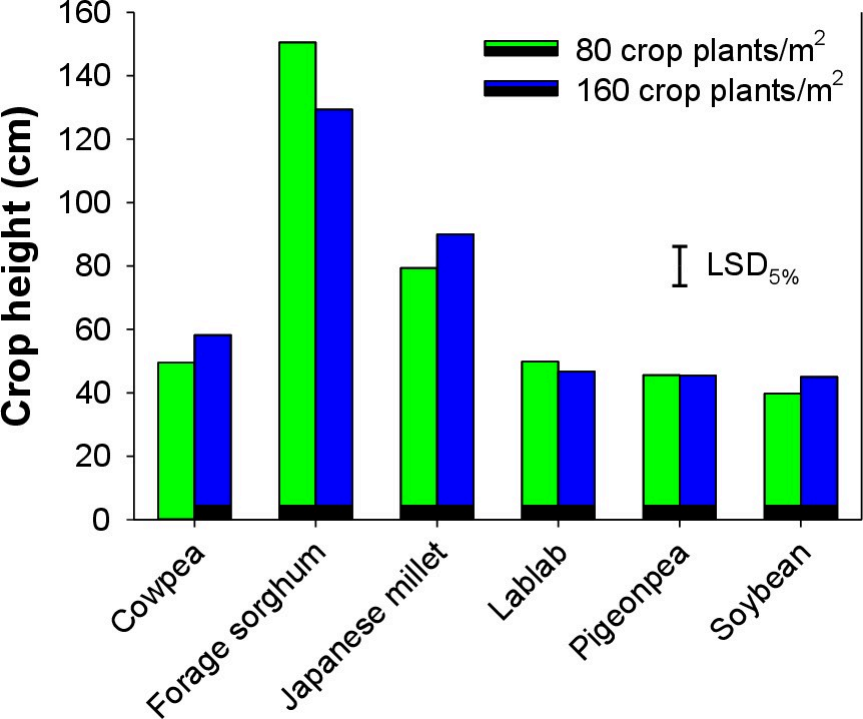
Plant height of various cover crops at planting densities of 80 and 160 plants m^-2^.

**Fig 8:**
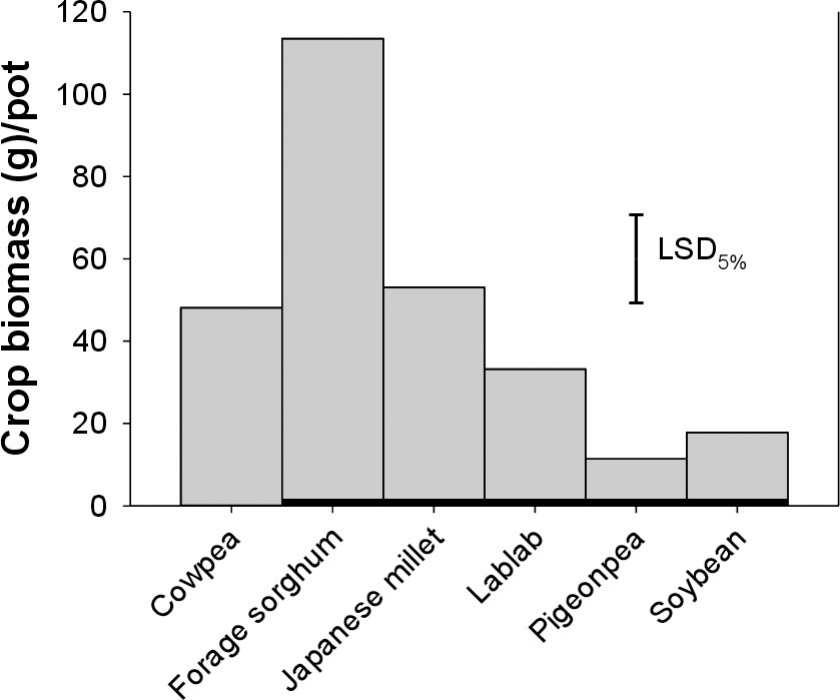
Biomass of various cover crops used in the present study.

Cowpea, lablab, pigeonpea, and soybean plants had similar plant height (Fig 7). The tallest cover crop plants were that of forage sorghum followed by Japanese millet. The height of forage sorghum was significantly higher than all other cover crop plants. The biomass of cover crops was not affected by their densities and they produced variable biomass ranging from as low as 11.4 g to 113.5 g pot^-1^. Pigeonpea and soybean recorded significantly lower plant biomass (11.4 and 17.8 g pot^-1^, respectively) than the rest of the cover crops used in the present study (Fig 8). Forage sorghum produced the highest plant biomass (113.5 g pot^-1^), and Japanese millet plants recorded the next higher biomass (53.1 g pot^-1^).

## Discussion

The presence of cover crops in the neighborhood of *E*. *colona* reduced the height, leaves, and tillering capacity of this weed. The reduction in weed growth attributes due to the cover crop interference is related to diverse mechanisms involving resource deprivation, modification in microclimate elements such as temperature and light regimes, moisture contents, and allelopathy [14, 19, 25]. Niche pre-emption has been proved as a prime mechanism conducive to suppressing the growth and development of weeds by cover crops [19]. Cover crops utilized space and growth resources that otherwise would have been utilized by *E*. *colona* plants. Live cover crops are more efficient compared to their residue in weed suppression [14]. Living cover crops absorb red light thereby reducing the red:far-red light ratio to modulate phytochrome-related responses [14] and cause shading which diminishes photosynthesis of weeds growing underneath cover crops. The reduction in seedling morphological attributes was translated into lower *E*. *colona* plant biomass due to reduced height, leaves, and tillers. *E*. *colona* plants utilized the available resources to their full benefits in the absence of cover crops and manifested tall plants with profuse tillering and numerous leaves. These observations suggest that this weed could be devastating during fallow periods due to its aggressive growth behavior.

Cover crops used in the present study caused variable suppression of *E*. *colona*. Leguminous cover crops, especially pigeonpea, were least effective in reducing seedling growth of this weed. The possible reason is their short stature and slow initial growth, due to which *E*. *colona* plants attained greater height than these cover crop plants. Additionally, due to their low C:N ratio, leguminous cover crops decompose quickly and offer less potential for weed suppression later in the growing season [26]. At harvest, *E*. *colona* plants were taller than all leguminous cover crops. On the other hand, cover crops like forage sorghum and Japanese millets with height greater than *E*. *colona* plants effectively suppressed seedling growth and biomass accumulation of this weed. When water and nutrient are non-limiting, plants principally compete for light and tall growing plants outcompete the shorter plants growing under their canopy. Rapid growth and development of a cover crop contribute towards its ability to close the canopy faster and cover the ground surface quickly and early in the season. Development of early-season ground cover was more valuable than total dry matter production by velvetbean accessions (*Mucuna cochinchinensis*, *M*. *pruriens* var. *utilis*, and *M*. *pruriens* var. IRZ) for *Imperata cylindrica* (L.) Raeuschel. suppression [27]. However, the present study suggested that cover crops with taller height and greater biomass accumulation are effective against *E*. *colona* compared to those with short height and less biomass.

A negative relationship between the biomass of cover crops and weeds has been documented [14, 28]. A recent study also reported lower biomass of leguminous cover crops such as cowpea than grasses like Sudan grass [*Sorghum × drummondii* (Steud.) Millsp. & Chase] [17]. Cover crops with greater biomass close their canopy early and provide greater suppression of associated weeds [29]. Cover crop-mediated weed suppression was poor when the growth of a cover crop was slow, and it failed to cover the ground completely [30]. The fact that forage sorghum as a cover crop suppressed *E*. *colona* to a greater extent could also be attributed to the ability of this plant to release allelochemicals in the environment, having the potential to hamper weed germination and establishment [31].

Increased crop density can contribute towards crop competitiveness against weeds. However, in the present study, the increased density of cover crops did not affect the height and biomass of *E*. *colona*. In a recent study, an increase in mungbean [*Vigna radiata* (L.) R. Wilczek] density from 164 plants m^-2^ to 246 plants m^-2^ increased the suppression magnitude of *Sonchus oleraceus* L. shoot biomass from 69% to 86% [32]. However, the suppressive effects of increased crop density on target weeds are species-specific. A previous study, for example, found that an increase in rice density negatively affected the height of *Ludwigia octovalvis* (Jacq.) Raven, while no such suppressive effect was observed on *Amaranthus spinosus* L [33]. In our study, emerging cover crop plants might have compensated their lower number by more growth per plant, so cover crop biomass at harvest was not affected by planting density. These results also suggest that a low density (e.g., 80 plants m^-2^) of cover crops will be sufficient to provide effective suppression of weeds.

Seed production is a vital attribute governing the weed seedbank and overall weed population dynamics under field conditions. Our results suggest that interference by cover crops like forage sorghum and Japanese millet can help in reducing *E*. *colona* growth and seed production and support the recommendation of a fast-growing, weed suppressive, and competitive cover crop to suppress weeds [14, 19]. A live cover crop can inhibit weed seed production, provided sufficient growth suppression [34]. Moreover, weed seed predation at the soil surface was also enhanced in the presence of a living cover crop [35]. Although *E*. *colona* seed production was significantly reduced and plants of this weed growing in interference with forage sorghum and Japanese millet produced only 300–400 seeds plant^-1^, this amount of seed output is enough to cause heavy infestations in years to come [36]. Nevertheless, compared to the enormous seed production of this weed (when grown alone), the use of cover crops seems promising to reduce contribution to the seedbank and at the same time warrants the need for additional control measures. Hence, it can be inferred that to manage weeds that are prolific seed producers, as is the case with *E*. *colona*, sole reliance on cover crops to suppress weeds is not viable in the backdrop of long-term weed management.

The use of forage sorghum and Japanese millets as vigorous ground cover crops during the fallow period will suppress weeds and provide much-needed improvement in soil quality. However, to harness the full benefits of agroecosystem services rendered by cover crops, fine-tuning is needed, and these should be used in conjunction with other weed control measures to have a broader impact on weeds with a low amount of seeds reaching the seedbank. These cover crops need to be further validated under field conditions to suppress *E*. *colona* and other problematic weeds. Cover crops grown as mixtures also need to be explored.
